# Tamoxifen Ameliorates Peritoneal Membrane Damage by Blocking Mesothelial to Mesenchymal Transition in Peritoneal Dialysis

**DOI:** 10.1371/journal.pone.0061165

**Published:** 2013-04-23

**Authors:** Jesús Loureiro, Pilar Sandoval, Gloria del Peso, Guadalupe Gónzalez-Mateo, Vanessa Fernández-Millara, Beatríz Santamaria, Maria Auxiliadora Bajo, José Antonio Sánchez-Tomero, Gonzalo Guerra-Azcona, Rafael Selgas, Manuel López-Cabrera, Abelardo I. Aguilera

**Affiliations:** 1 Centro de Biología Molecular-Severo Ochoa, CSIC-UAM, Cantoblanco, Madrid, Spain; 2 Servicio de Nefrología, Hospital Universitario La Paz, Instituto de Investigación Sanitaria la Paz (IdiPAZ), Madrid, Spain; 3 Unidad de Diálisis and Laboratorio de Investigación Renal y Vascular, Fundación Jiménez Díaz, Madrid, Spain; 4 Unidad de Biología Molecular and Servicio de Nefrología, Hospital Universitario de la Princesa, Instituto de Investigación Sanitaria Princesa (IP), Madrid, Spain; 5 Servicio de Cirugía, Hospital Quirón San Camilo, Madrid, Spain; University of Louisville, United States of America

## Abstract

Mesothelial-to-mesenchymal transition (MMT) is an auto-regulated physiological process of tissue repair that in uncontrolled conditions such as peritoneal dialysis (PD) can lead to peritoneal fibrosis. The maximum expression of peritoneal fibrosis induced by PD fluids and other peritoneal processes is the encapsulating peritoneal sclerosis (EPS) for which no specific treatment exists. Tamoxifen, a synthetic estrogen, has successfully been used to treat retroperitoneal fibrosis and EPS associated with PD. Hence, we used *in vitro* and animal model approaches to evaluate the efficacy of Tamoxifen to inhibit the MMT as a trigger of peritoneal fibrosis. *In vitro* studies were carried out using omentum-derived mesothelial cells (MCs) and effluent-derived MCs. Tamoxifen blocked the MMT induced by transforming growth factor (TGF)-β1, as it preserved the expression of E-cadherin and reduced the expression of mesenchymal-associated molecules such as snail, fibronectin, collagen-I, α-smooth muscle actin, and matrix metalloproteinse-2. Tamoxifen-treatment preserved the fibrinolytic capacity of MCs treated with TGF-β1 and decreased their migration capacity. Tamoxifen did not reverse the MMT of non-epitheliod MCs from effluents, but it reduced the expression of some mesenchymal molecules. In mice PD model, we demonstrated that MMT progressed in parallel with peritoneal membrane thickness. In addition, we observed that Tamoxifen significantly reduced peritoneal thickness, angiogenesis, invasion of the compact zone by mesenchymal MCs and improved peritoneal function. Tamoxifen also reduced the effluent levels of vascular endothelial growth factor and leptin. These results demonstrate that Tamoxifen is a therapeutic option to treat peritoneal fibrosis, and that its protective effect is mediated via modulation of the MMT process.

## Introduction

In peritoneal dialysis (PD), the peritoneal membrane (PM) is exposed to bio-incompatible dialysis solutions, with high content of glucose, which can cause peritoneal injury when associated with peritoneal incidents like repeated episodes of peritonitis or hemoperitoneum [1], [2], [3]. Progressive fibrosis, angiogenesis and ultimately, ultrafiltration failure, are some characteristics of the so-called sclerotic peritonitis syndromes [4]. Several pathologic factors, such as inflammatory mediators, high glucose content, the presence of glucose degradation products, and low pH can induce peritoneal mesothelial cells (MCs) to lose certain epithelial characteristics and progressively acquire a fibroblast-like phenotype soon after initiation of PD. This so-called mesothelial-to-mesenchymal transition (MMT) serves as a trigger for peritoneal fibrosis and angiogenesis, via up-regulation of transforming growth factor (TGF)-β1 and vascular endothelial growth factor (VEGF), respectively [5], [6]. As such, MMT is considered an important potential therapeutic target in peritoneal deterioration [7], [8].

Encapsulating peritoneal sclerosis (EPS) is a severe form of peritoneal fibrosis characterized by intestinal encapsulation through the deposition of excessive matrix components that subsequently may lead to obstruction of the intestinal tract. Although rare, EPS is a serious complication of PD for which no specific and definitive treatment exists. However, peritoneal resting, steroids, immunosuppressive agents and Tamoxifen have been used previously as therapeutic approaches with divergent results [9], [10], [11], [12]. Tamoxifen is an estrogen receptor modulator used for the treatment of breast cancer [13]. Tamoxifen can also affect the activity of TGF-β1 and has been shown to be effective in fibrotic diseases as retroperitoneal fibrosis. In this context, in 1991 Clark et al. reported a dramatic reduction of peritoneal fibrosis and mortality in two patients diagnosed with retroperitoneal fibrosis and treated orally with Tamoxifen [14]. Given the high morbidity and mortality associated with EPS, the lack of specific treatments, and the therapeutic potential of Tamoxifen [14], in 1992 we started the first clinical study to analyze the effects of oral Tamoxifen treatment (20 mg every 12 h) in PD patients suffering this peritoneal complication. Their evolution was compared with a historic EPS control group collected between 1980 and 1992. We found a significant reduction in surgical abdominal complications, hospital admission rates and mortality in comparison with the non-treated patients [15]. Similar results were obtained in other clinical studies using Tamoxifen to treat EPS [16].

These clinical experiences and the information provided by other investigators in regard to the anti-fibrotic and anti-angiogenic effects associated to Tamoxifen-treatments [16], [17], [18], [19] encouraged us to study the molecular mechanisms involved in the peritoneal protective effects of Tamoxifen in more detail. Thus, we have specifically analyzed the effect of Tamoxifen on the MMT of MCs, both *in vitro* and in an animal experimental model, given the central role of this process in the initiation and progression of peritoneal injury in PD patients [5], [7], [8].

We found that Tamoxifen blocked and reverted the MMT of MCs *in vitro* and partially reverted the mesenchymal characteristics of effluent-derived MCs. In mice exposed to PD fluid, Tamoxifen ameliorated peritoneal thickness and angiogenesis, and decreased submesothelial MMT.

## Materials and Methods

### Culture of omentum and effluent-derived MCs and treatments

MCs were obtained from omental samples taken from patients undergoing elective abdominal surgery and from the effluents of PD patients as described previously [5], [20], [21]. The purity of the omentum- and effluent-derived MCs cultures was determined by the expression of the standard mesothelial markers: intercellular adhesion molecule (ICAM)-1, calretinin and cytokeratins. These MCs cultures were negative for von-Willebrand factor and CD45, ruling out any contamination by endothelial cells or macrophages [5], [20], [21]. To induce MMT *in vitro*, omentum-derived MCs were seeded on wells coated with collagen I (50 µg/mL, Roche Diagnostics GmbH, Mannheim, Germany) and treated for different time points (12 to 48 hours) with human-recombinant TGF-β1 (1 ng/mL, R&D Systems Inc, Minneapolis, MN.), a commonly used *in vitro* model of MMT [5], [22], [23], [24]. Where indicated Tamoxifen (Lilly Research Laboratories, Indianapolis, Indiana, USA) was administered at concentrations of 3 and 6 µM, as has been referred by others [25], [26], [27], [28]. Effluent-derived MCs that have undergone a MMT (as determined by non-epitheliod morphology, by low expression of E-cadherin and by up-regulated expression of mesenchymal markers) were also administrated with different doses of Tamoxifen (3, 6, and 10 µM) and analyzed at 48 hours.

To evaluate the ability of Tamoxifen to revert the MMT *in vitro*, omentum-MCs were stimulated with TGF-β1 during 48 hours and then subdivided into four groups. In group 1, MCs were treated with TGF-β1 for additional 48 hours (total 96 hours). In group 2, TGF-β1 was withdrawal and the cells were left untreated during the next 48 hours to observe the spontaneous evolution of MMT. In the groups 3 and 4, the cells were treated with Tamoxifen (6 or 10 µM), after TGF-β1 withdrawal, during 48 hours. At the end of experiment, MCs were morphologically analyzed and the expression of E-cadherin was determined by quantitative RT-PCR.

The present study adjusts to the Declaration of Helsinki and it was approved by the Ethic Committee of “Hospital Universitario de la Princesa” (Madrid, Spain). Informed written consent was obtained from all of the patients (PD effluent and omentum donors).

### Western blot, quantitative RT-PCR, enzyme-linked immunoassays, and migration assays

For western blotting, MCs cultures were lysed in a buffer containing 1% sodium deoxycholate and 0.1% sodium dodecyl sulfate (SDS). The total protein was quantified using a protein assay kit (Bio-Rad, Hercules, CA). Total cell protein (50 µg) was resolved on 8–10% SDS-polyacrylamide gels and transferred to nitrocellulose membranes, which were then blocked with fat-free milk and probed with specific antibodies against α-ER (Santa Cruz Biotechnology Inc, Santa Cruz, CA), E-cadherin, α-SMA, collagen I, fibronectin, MMP-2 and tubulin (Sigma-Aldrich, Inc, St Louis MO). These antibodies were detected with a peroxidase conjugated goat anti-mouse IgG antibody (BD Biosciences, Franklin Lakes, NJ) and visualized by enhanced chemilumniscence (ECL detection kit, Amersham Biosciences, Freiburg, Germany). Images of the blots were acquired with a LAS-1000 Charge Coupled Device camera (Fujifilm, Cedex, France).

For quantitative RT-PCR analysis, MCs were lysed in TRI Reagent (Ambion Inc, Austin, TX), and RNA was extracted as fabricant instructions. Complementary-DNA was synthesized from 2 µg of total RNA by reverse transcription (RNA PCR Core Kit, Applied Biosystems Inc, New Jersey). Quantitative PCR was carried out in a Light Cycler 2.0 using a SYBR Green Kit (Roche Diagnostics GmbH) and specific primers sets for Snail, E-cadherin and histone H3. Samples were normalized with respect to the value obtained for H3. The primer sets employed for Snail, E-cadherin and H3 have been previously described [22].

To evaluate the fibrinolytic capacity of MCs isolated from omentum and from PD effluent, the concentration of plasminogen activator inhibitor type-1 (PAI-1), tissue-type plasminogen activator (tPA), urokinase-type plasminogen activator (uPA) and urokinase-type plasminogen activator receptor (uPAR) was measured in culture supernatants by ELISA kits (R&D Systems Inc).

To study migration, 5×10^4^ MCs that were either treated or not with TGF-β1 and different doses of Tamoxifen for 24 hours, were re-suspended in 100 µL of M199 supplemented with 0.1% human serum albumin (HSA) and plated in the upper chamber of polycarbonate membrane transwell units (6.5 mm diameter, 5 µm-diameter pore size; Costar, Corning, NY), while 600 µL of M199 with 0.1% HSA and recombinant epidermal growth factor (10 ng/mL) was placed in the lower chamber. MCs were allowed to migrate for 24 hours in an atmosphere of 5% CO_2_ at 37°C. The cells that had migrated were recovered from the underside of the membrane, detached with trypsin-EDTA (0.05%), washed and resuspended in M199 medium. Finally, the cells were stained with propidium iodide and counted over 1.5 min by flow cytometry (FACS Canto II cytometer; BD Biosciences).

### Effect of Tamoxifen on cell cycle, apoptosis, and wound healing

Given that the regulation of estrogen receptor has been implicated in cell proliferation and apoptosis [29], [30] we analyzed if Tamoxifen affected the cell cycle and apoptosis in MCs. To analyze the effect of Tamoxifen on cell cycle profile, omentum MCs were grown with fetal calf serum 20% and treated with different doses of Tamoxifen (3, 6, and 10 µM) for 48 h. Cells were trypsinized, pelleted, and fixed with 70% cold ethanol for 30 min. After washing, samples were suspended in PBS and an equal volume of propidium iodide solution, containing 200 µg/mL RNase (Sigma Aldrich), 20 µg/mL propidium iodide (Sigma Aldrich), and 0.1% Triton X-100 in PBS, was added to the cell suspension for 30 min at room temperature. A FACS Calibur flow cytometer (BD Bioscience) was used to analyze DNA content; emitted light was measured at 675 nm.

To analyze the effect of Tamoxifen on apoptosis, MCs were cultured to subconfluence in 12-well plates for 24 hours. Cells were pre-incubated with Tamoxifen (3, 6, and 10 µM) and treated with 100 nM Staurosporine (STS) during 24 hours [31]. For quantification of cell death, cells were harvested by pooling non-adherent cells with adherent cells, which were detached by gentle trypsinization. Apoptosis was quantified by flow cytometry assessment of DNA content using Cellquest software (BD Biosciences).

To test the effect of Tamoxifen on wound repair capacity of MCs, a wound healing experiment was performed with cells treated or not with different doses of Tamoxifen (3 and 6 µM). Confluent culture of MCs from omentum were subjected to mechanical injury with an adapted cell scraper approximately 1500 µm in width and photographed every twelve hours during 72 hours.

### Peritoneal dialysis fluid exposure model in mice

A total of 61 female C57BL/6 mice aged between 12 and 16 weeks old were used in this study (Charles River S.A., Barcelona, Spain). The experimental protocol used was in accordance with the National Institutes of Health Guide for Care and Use of Laboratory Animals and was approved by the Animal Ethics Committee of the “Unidad de Cirugía Experimental” of “Hospital Universitario la Paz”. PD fluid or saline solution were instilled via a peritoneal catheter connected to an implanted subcutaneous mini access port (Access Technologies, Skokie, IL, USA) as previously described [22], [23].

During the first week after surgery, the animals implanted with a peritoneal access port received 0.2 mL of saline with 1 IU/mL heparin. Thereafter, during a 4-week period, 10 mice were daily instilled with 1.5 mL of saline solution (Control group), 17 mice were daily instilled with 1.5 mL of standard PD fluid (PDF group) composed of 4.25% glucose and buffered with lactate (Stay Safe; Fresenius, Bad Homburg, Germany) and 19 mice were treated with oral Tamoxifen [32], [33] and daily instilled with 1.5 mL of standard PD fluid (PDF + Tamoxifen group).

Tamoxifen was diluted in water (1.5 mg/kg/day in 15 µL of volume). This volume was orally administered to mice using a pipette and a gastric tube. Three animals of the PDF group, two from PDF + Tamoxifen group and one from Control group were not used in the final analysis, being the main causes of drop-outs catheter port infection or traumatic catheter removal (Control group, n = 9; PDF group, n = 14 and PDF + Tamoxifen group, n = 17). A peritoneal equilibrium test was performed during the last day of treatments. Mice were instilled with 2 mL of PD solution and after 30 min, animals were anaesthetized with isoflurane (MTC Pharmaceuticals, Cambridge, ON, Canada) and sacrificed to recover the total peritoneal volumes [22], [23]. Parietal peritoneum pieces were obtained from the contralateral side of the implanted catheter. Food and water were provided *ad libitum* to the animals.

To evaluate the time course progression of MMT and peritoneal fibrosis, we made an additional experiment in which 15 mice were daily instilled with 1.5 mL of standard PD fluid and sacrificed at 7, 15, or 30 days; five mice were included in each time point. Submesothelial MMT markers and peritoneal thickness were measurement as described below.

### Histological analysis of peritoneal samples and effluent growth factors measurement

For histochemical analysis, parietal peritoneum tissues were routinely fixed in Bouin's liquid and embedded in paraffin to obtain serial tissue sections 3–4 µm thick. Deparaffinized sections were stained with Masson's trichrome solution to analyze the histological characteristics of each specimen. The thickness of submesothelial tissue was determined by blinded microscope analysis using a metric ocular, and was expressed as the mean of 10 independent measurements for each animal.

For immunofluorescence analysis, cryostat sections (5 µm) were stained with antibodies to visualize vasculature (CD31; Serotec, Oxford, UK), mesothelial cells (Pan-Cytokeratin; Sigma-Aldrich), and pathologic fibroblasts (FSP1; Dako). The frozen sections were fixed for 15 minutes in 4% formaldehyde in PBS, and blocked with 10% horse serum for 1 hour in PBS with 0.3% Triton X-100. First antibodies were incubated in PBS with 0.1% Triton X-100 overnight at 4°C. After 3 washing steps, secondary Alexa-labelled antibodies were incubated during 90 minutes at room temperature. After another washing process, the preparations were mounted with a 4,6-diamidino-2-phenylindole (DAPI) nuclear stain (Vectashield; Vector Laboratories). Negative controls for immunofluorescence staining were conducted using 10% rabbit serum instead of primary antibody. Images were analysed by computerized digital image analysis (AnalySIS, Soft Imaging System). Number of cells with single or double positive staining was counted and was expressed as the mean of 10 independent measurements for each animal [22].

The amounts of VEGF, TGF-β1 and Leptin in the peritoneal effluents were determined by ELISA-based assays, according to the manufacturers instructions (Bender MedSystems, Vienne, Austria; BD Biosciences Pharmingen, San Diego, CA; and Mediagnost, Reutlingen, Germany, respectively).

### Statistical analysis

Results are presented as mean ± SE in bars graphics and as 25th and 75th percentiles, median, minimum, and maximum values in box plot graphics. Lineal regression analysis was used to evaluate the correlation between submesothelial MMT and peritoneal thickness (Spearman test). The data groups were compared with ANOVA one way and Mann–Whitney rank sum U-test using the SPSS statistic package version 14.5 (Chicago, IL) and GraphPad Prism version 4.0 (La Jolla, CA). P<0.05 was considered statistically significant.

## Results

### Tamoxifen blocks and reverts MMT *in vitro* and partially reverts the mesenchymal phenotype of effluent-derived MCs

**Figure 1:**
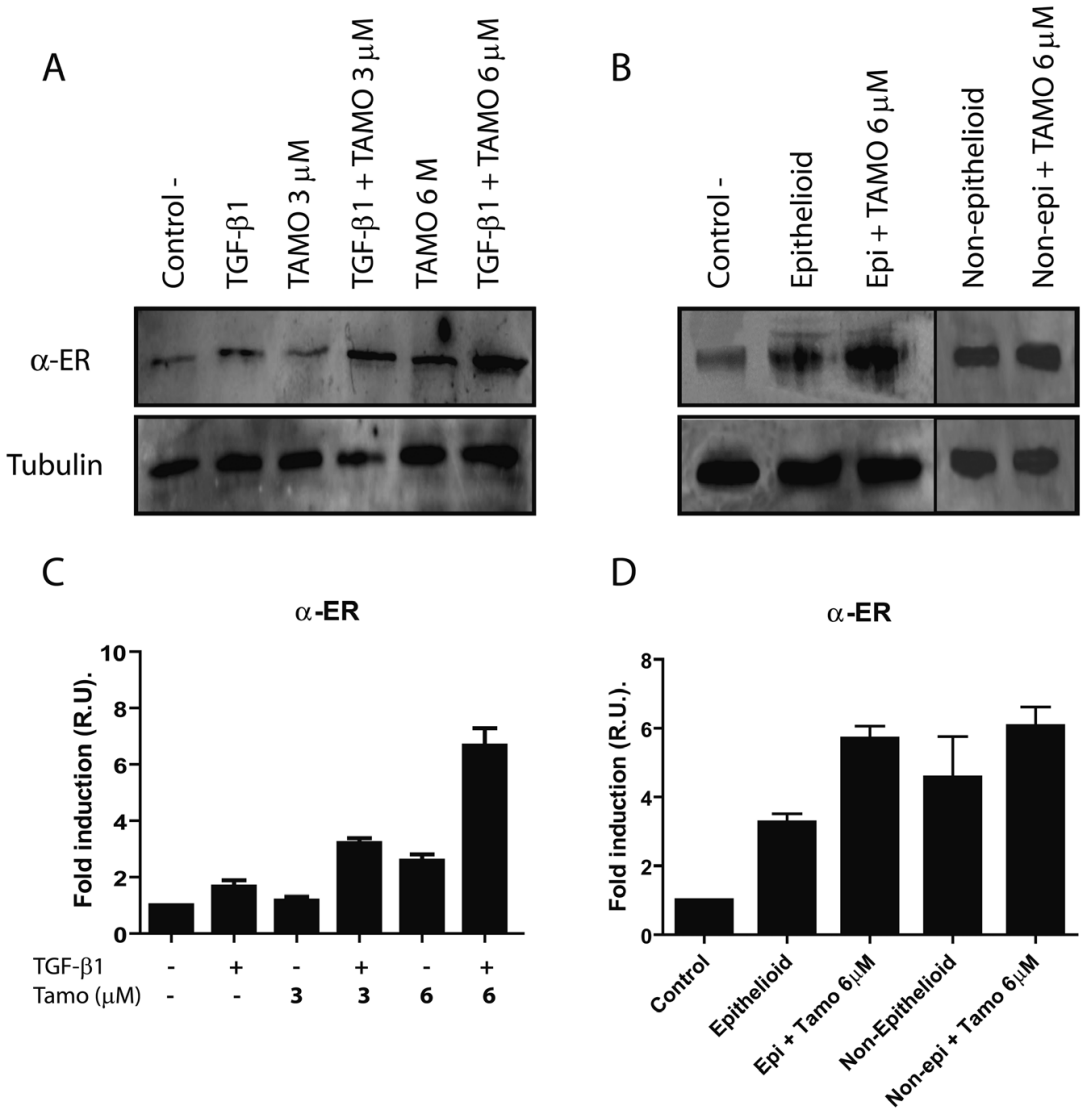
Tamoxifen increases α-ER expression in MCs isolated from omentum and PD effluent. Omentum-derived MCs were treated or not for 24 hours with TGF-β1 in the presence of different doses of Tamoxifen (0, 3 and 6 µM). (**A**) Western blot analysis shows that MCs express α-ER and that its expression is up-regulated by Tamoxifen in cooperation with TGF-β1. (**B**) Epithelioid and non-epithelioid MCs isolated from PD effluent showed up-regulation of α-ER expression. After Tamoxifen 6 µM administration, both group of cells showed a further increase of α-ER expression. A representative experiment is shown. (**C and D**) Bars in graphic represent means ± SE of three independent experiments.

We first analyzed whether omentum-derived MCs expressed estrogen receptor alpha (α-ER), and how its expression was affected by TGF-β1 and/or different doses of Tamoxifen (3 and 6 µM). Tamoxifen increased the expression of α-ER in a dose-dependent manner and in cooperation with TGF-β1; a six-fold increase of α-ER expression was observed. Similarly, epithelioid and non-epithelioid MCs from PD effluents showed up-regulated expression of α-ER that further increased upon Tamoxifen administration (Figure 1). Then we analyzed if Tamoxifen affected the cell cycle/apoptosis and the wound repair capacity of MCs. The cells did not display significant differences in cell cycle between control and Tamoxifen treatments (Table S1). Moreover, Tamoxifen protected MCs from apoptotic death induced by Staurosporine. These results indicated that Tamoxifen was not cytotoxic for MCs and that rather, it produced an anti-apoptotic effect in these cells (Figure S1). Tamoxifen treatment did not influence significantly wound healing capacity of MCs (Figure S2).

**Figure 2:**
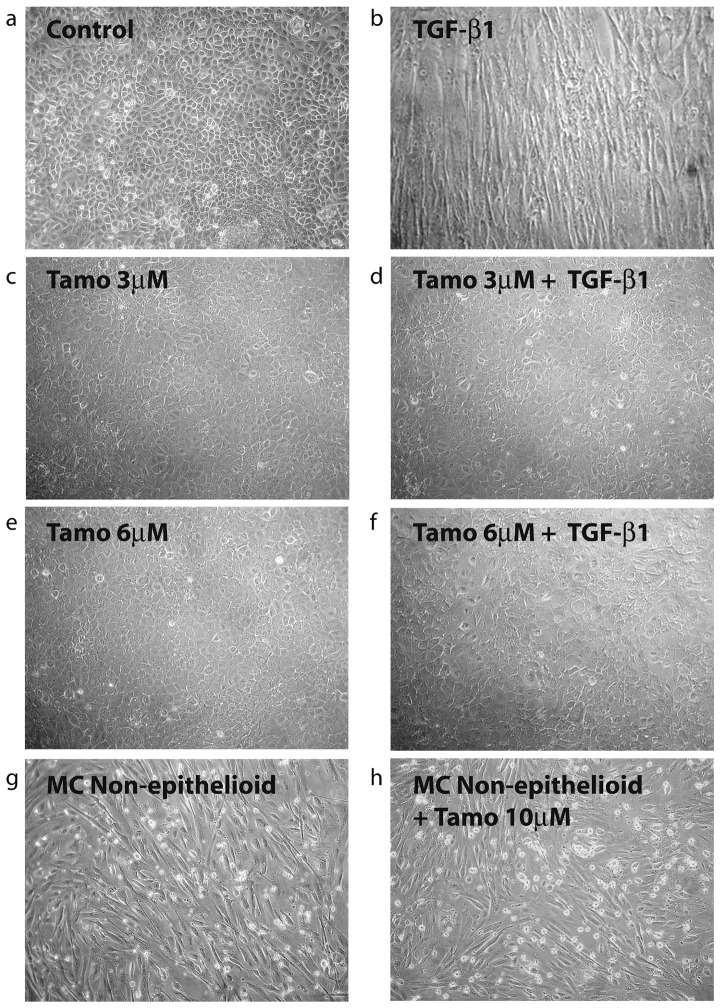
Tamoxifen blocks the TGF-β1-induced MMT but does not revert the mesenchymal morphology of effluent-derived MCs. (a to f) Omentum-derived MCs were treated or not for 48 hours with TGF-β1 in the presence of different doses of Tamoxifen (0, 3 and 6 µM). Phase-contrast microscopy shows that Tamoxifen treatment prevents the morphological change induced by TGF-β1. (g and h) Effluent-derived MCs with non-epithelioid morphology were either left untreated or treated with Tamoxifen (10 µM) during 48 hours. Phase-contrast microscopy shows that Tamoxifen does not revert the morphology of MCs to an epithelioid phenotype.

**Figure 3:**
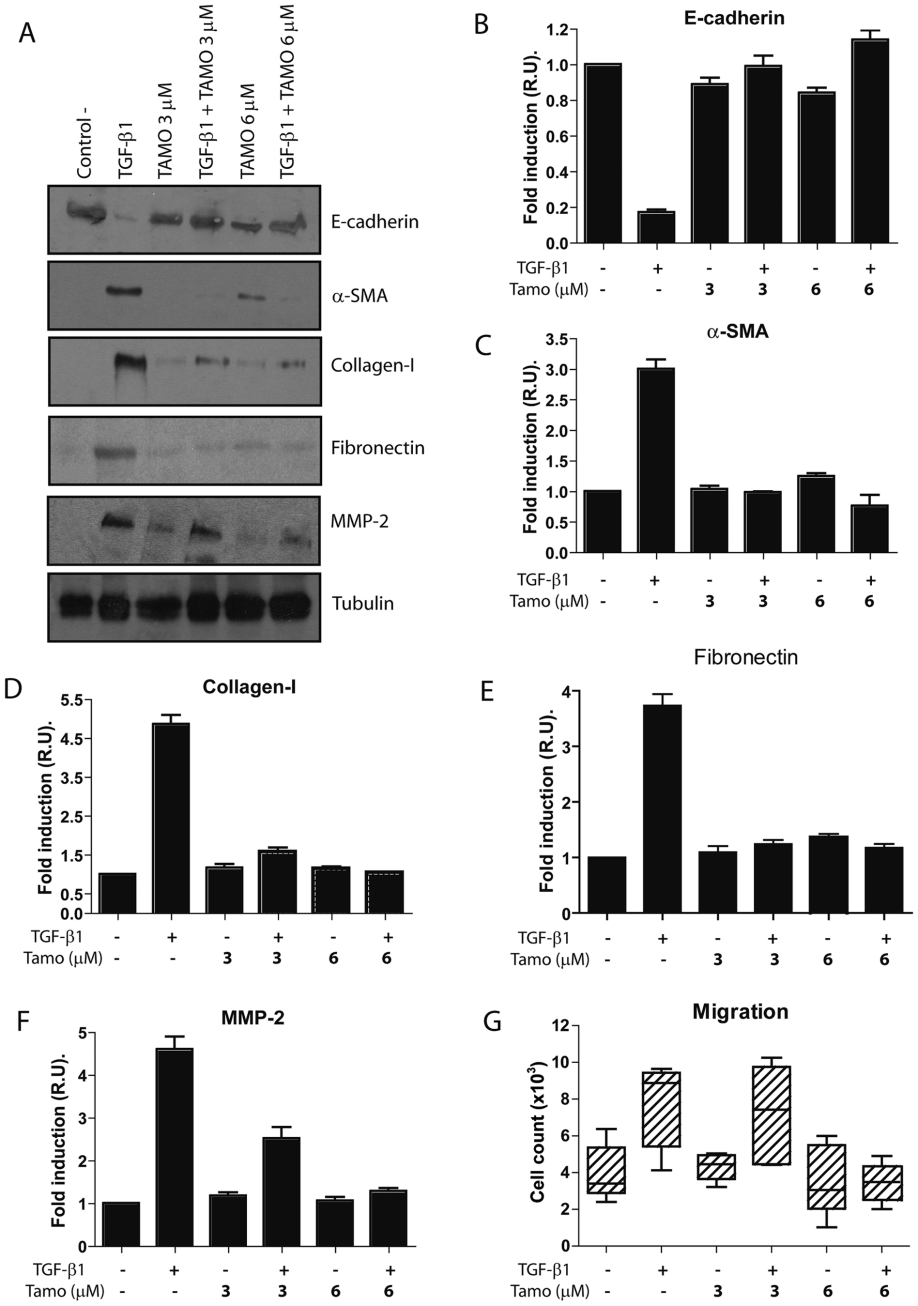
Tamoxifen blocks TGF-β1-induced MMT of MCs. Omentum-derived MCs were treated or not with 1 ng/mL TGF-β1 for 24 or 48 hours, in the presence of different doses of Tamoxifen (0, 3 and 6 µM). (**A**) Western blot analyses show that Tamoxifen treatment prevents TGF-β1-induced E-cadherin down-regulation as well as α-SMA, collagen I, fibronectin and MMP-2 up-regulation. A representative experiment is shown. (**B to F**) The experiments were repeated at least five times and results are depicted as means ± SE. The expressions of E-cadherin (**B**) and MMP-2 (**F**) were analyzed at 24 hours, whereas the expressions of α-SMA (**C**), collagen I (**D**) and fibronectin (**E**), were analyzed at 48 hours of treatments. (**G**) Analysis of the migration capacity in transwell units demonstrates that Tamoxifen (6 µM) reduces the TGF-β1-indued migratory capacity of MCs to basal levels. The experiments, made in triplicates, were repeated at least four times. Box plots represent the median, minimum and maximum values, as well as the 25th and 75th percentiles.

Given the anti-fibrotic effects of Tamoxifen in retroperitoneal fibrosis and EPS and that the MMT of MCs is an important process in peritoneal deterioration we examined the effect of Tamoxifen on MMT of MCs. Treatment of omentum MCs with Tamoxifen (3 and 6 µM) blocked the TGF-β1-induced morphological change (Figure 2) and down-regulation of E-cadherin (Figures 3A and 3B). In addition, Tamoxifen interfered the TGF-β1-mediated up-regulation of MMT-associated proteins including α-smooth muscle actin (α-SMA), the matrix components collagen I and fibronectin, and matrix metalloproteinse-2 (MMP-2) (Figures 3C to 3F). During the MMT process the MCs acquire increased migratory capacity, thus we explored the effect of Tamoxifen on TGF-β1-induced migration. As shown in Figure 3G, Tamoxifen (6 µM) reduced the migratory capacity of MCs treated with TGF-β1 to basal levels.

**Figure 4:**
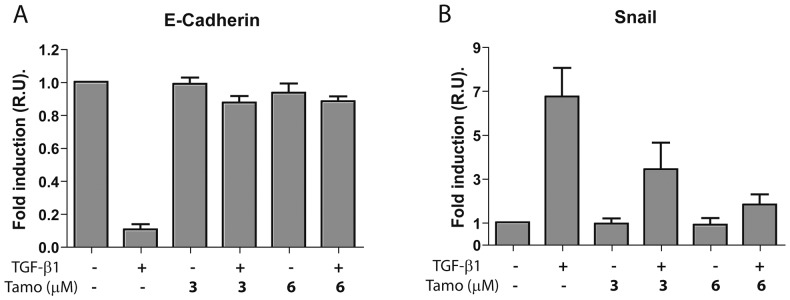
Tamoxifen interferes with TGF-β1-induced E-cadherin mRNA down-regulation and blocks the induction of Snail mRNA. Omentum-derived MCs were treated or not with 1 ng/mL TGF-β1 for 12 or 24 hours, in the presence of different doses of Tamoxifen (0, 3 and 6 µM). **(A)** Quantitative RT-PCR demonstrates that Tamoxifen treatments prevent TGF-β1-induced E-cadherin mRNA down-regulation. **(B)** Quantitative RT-PCR shows that Tamoxifen blocks TGF-β1-mediated induction of Snail mRNA, the main transcriptional repressor of E-cadherin. The expression of E-cadherin mRNA was analyzed at 24 hours, whereas the expression of Snail mRNA was analyzed at 12 hours of treatments. Bars depict means ± SE of three independent experiments.

To further demonstrate the MMT-blocking properties of Tamoxifen, we analyzed the effect of this drug on the transcription of E-cadherin-encoding mRNA. Tamoxifen treatments (3 and 6 µM) prevented TGF-β1-induced E-cadherin mRNA down-regulation (Figure 4A). Conversely, Tamoxifen blocked the induction by TGF-β1 of Snail mRNA, the main transcriptional repressor of E-cadherin (Figure 4B).

**Figure 5:**
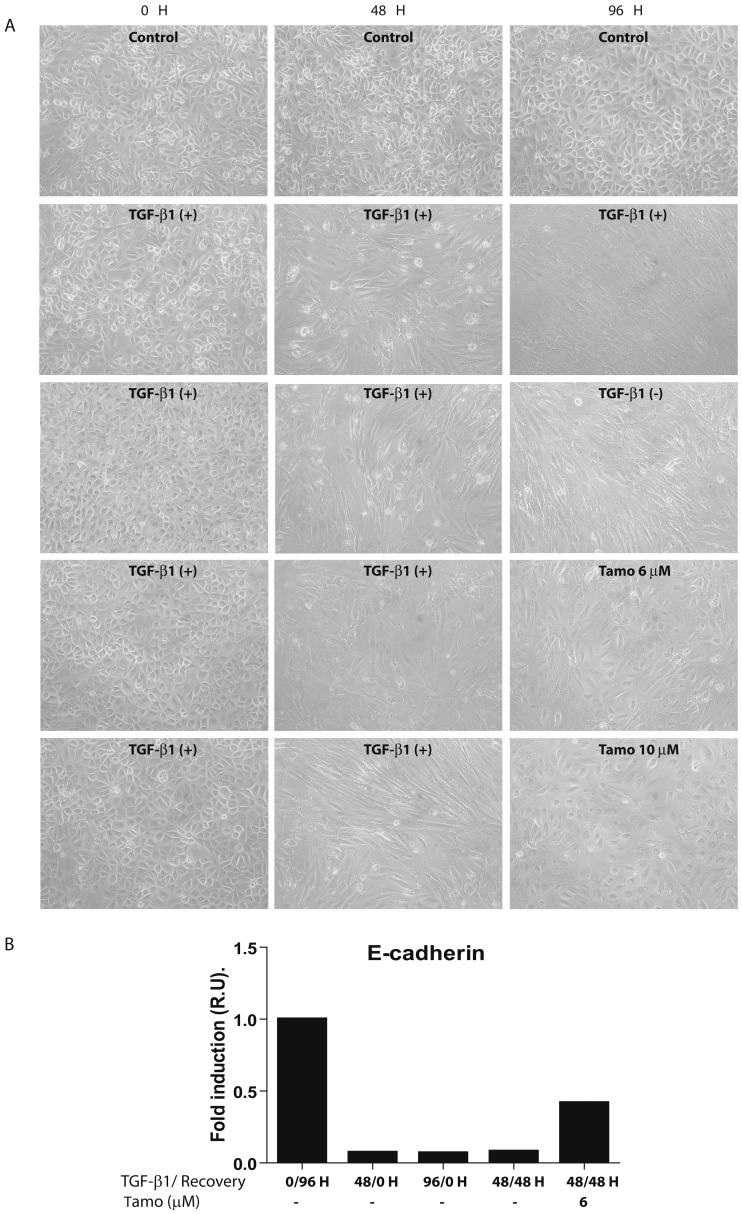
Tamoxifen reverts the MMT induced by TGF-β1 *in vitro*. Omentum-MCs were stimulated with TGF-β1 during 48 hours and then the cells were either left untreated, treated with TGF-β1 or treated with Tamoxifen (6 or 10 µM) during additional 48 hours. **(A)** Phase-contrast microscopy shows that Tamoxifen reverts partially the non-epithelioid morphology of omentum-derived MC treated with TGF-β1. **(B)** Quantitative RT-PCR analysis demonstrates that administration of Tamoxifen (6 µM) after TGF-β1 withdrawal restores partially E-cadherin expression. Bar graphic depicts the expression of E-cadherin-encoding mRNA in relative units (R.U.)

To analyze whether Tamoxifen was able to revert the MMT *in vitro*, omentum-MCs were stimulated with TGF-β1 during 48 hours and then the cells were either left untreated or treated with Tamoxifen (6 or 10 µM) during the next 48 hours. A group subjected to TGF-β1 stimulation for 96 hours was also included. The cells subjected to TGF-β1 stimulation for 48 and 96 hours showed a pronounced fibroblast-like shape and down regulation of E-cadherin (Figures 5A and 5B). After TGF-β1 withdrawal, the recovery of the epithelioid morphology was more evident in cells treated with Tamoxifen (6 or 10 µM) than in cells left untreated (Figure 5A). In addition, there was a partial re-expression of E-cadherin in cells treated with Tamoxifen (6 µM) after TGF-β1 withdrawal but not in cells left untreated (Figure 5B).

**Figure 6:**
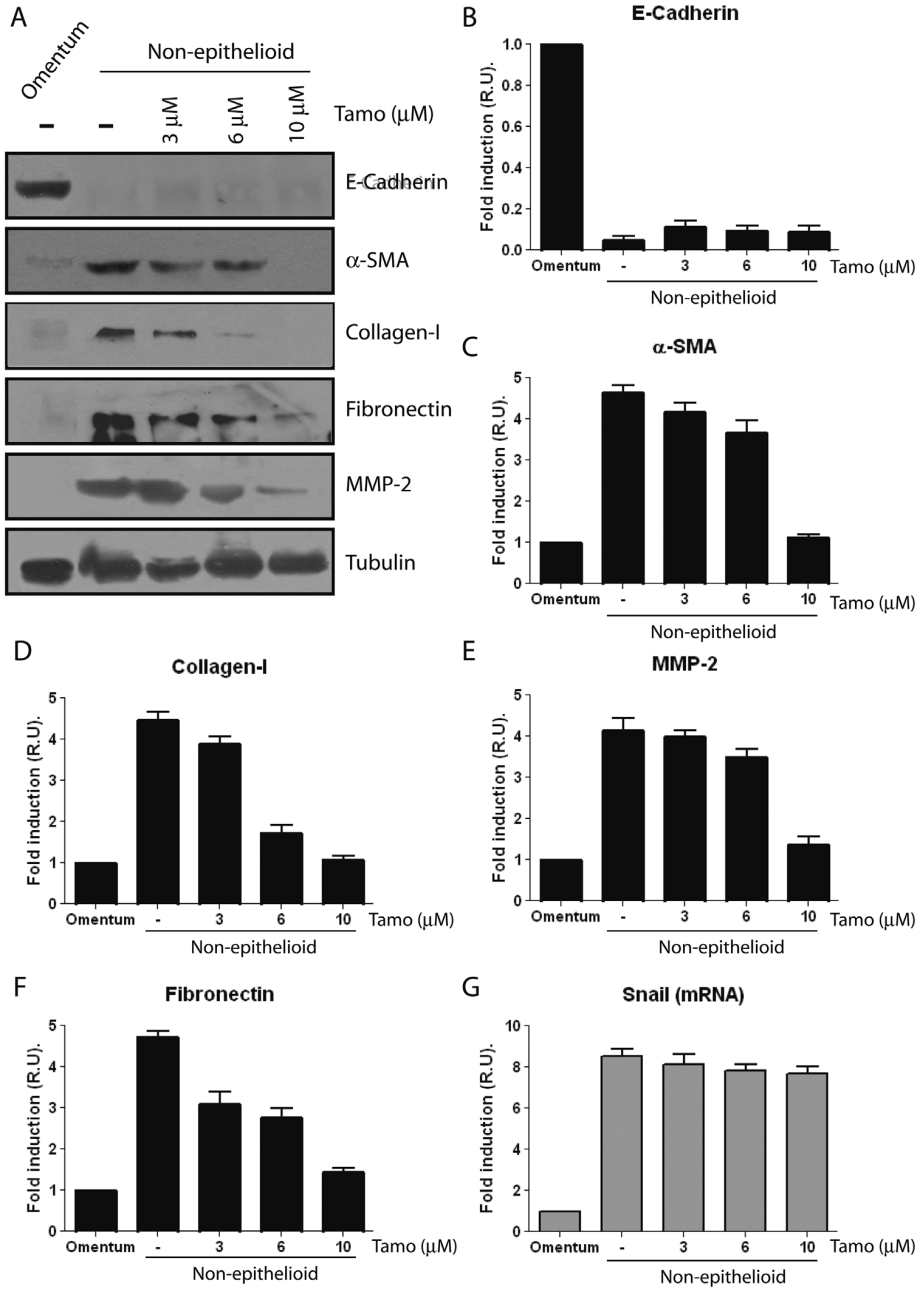
Tamoxifen reverts partially the mesenchymal phenotype of effluent-derived MCs. Effluent-derived MCs with mesenchymal phenotype (as determined by non-epitheliod morphology, low expression of E-cadherin and up-regulated expression of mesenchymal markers) were treated with different doses of Tamoxifen (0, 3, 6, and 10 µM) and analyzed at 48 hours. Omentum-derived MCs were employed as control. (**A**) Western blot analyses show that Tamoxifen treatments do not re-induce E-cadherin expression but inhibit the expression of the mesenchymal molecules α-SMA, collagen I, fibronectin and MMP-2; being the effects of Tamoxifen more evident at high doses (6 and 10 µM). A representative experiment is shown. (**B to F**) The experiments were repeated with five different samples of effluent-derived MCs and results of the expression of E-cadherin **(B)**, α-SMA **(C)**, collagen I **(D)**, MMP-2 **(E)** and fibronectin **(F)** are depicted as means ± SE. Quantitative RT-PCR demonstrates that the expression of Snail mRNA is not inhibited by any dose of Tamoxifen tested **(G)**. Bars depict means ± SE of five independent experiments.

By contrast, Tamoxifen treatments were unable to revert the spindle-like shape of effluent-derived MCs to an epithelioid morphology (Figure 2) and to re-induce the expression of E-cadherin (Figures 6A and 6B) even at high doses, 6 and 10 µM, of the drug. However, the expression of certain MMT-associated molecules including α-SMA, collagen I, fibronectin, and MMP-2 could be down-regulated by Tamoxifen, but only at high doses of the drug (Figures 6C to 6F). In agreement with the lack of effect of Tamoxifen on the expression of E-cadherin, this drug was unable to inhibit the expression of Snail-encoding mRNA at any concentration tested (Figure 6G).

### Tamoxifen preserves the fibrinolytic property of MCs treated with TGF-β1

**Figure 7:**
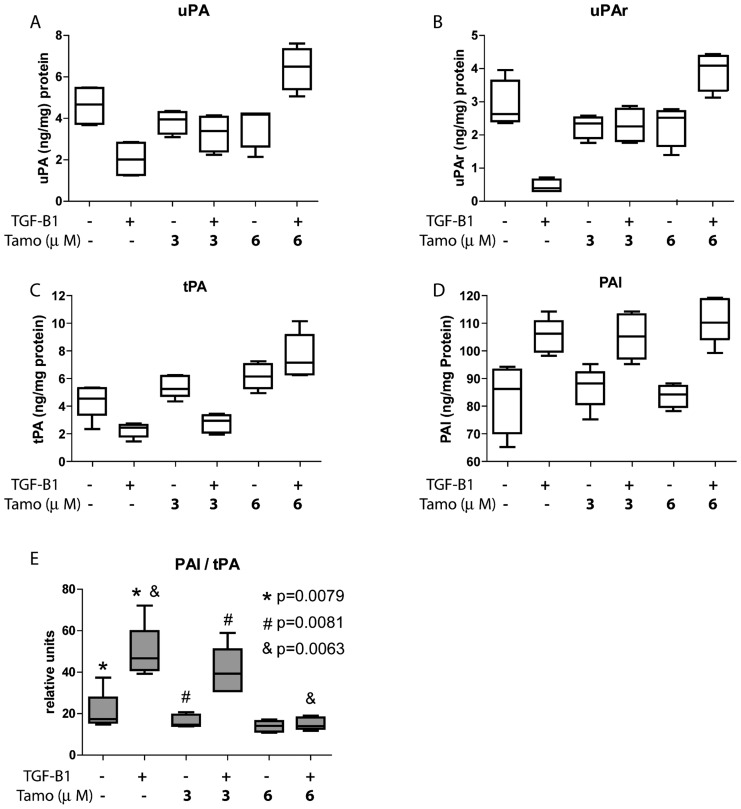
Tamoxifen preserves the fibrinolytic capacity of TGF-β1-treated MCs. Omentum-derived MCs were treated or not with 1 ng/mL TGF-β1 during 48 hours, in the presence of different doses of Tamoxifen (0, 3 and 6 µM). **(A to C)** Stimulation of MCs with TGF-β1 inhibits the expression of the fibrinolytic factors uPA **(A)**, uPAR **(B)** and tPA **(C)**, and treatments with different doses of Tamoxifen restore the basal levels of these factors or increase their synthesis above basal levels. **(D)**. TGF-β1 treatment increases the expression of PAI-1, and its expression is not affected by Tamoxifen. The levels of these factors were measured in culture media supernatants by ELISA and results are depicted as nanograms per milligrams of total cellular proteins **(E)**. The PAI/tPA-ratio, an important marker of fibrinolytic capacity decline, increases in response to TGF-β1 and returns to basal levels when Tamoxifen is added at 6 µM. Box plots show the 25th and 75th percentiles, median, minimum and maximum values of five independent experiments. The symbols represent the statistical differences between the groups.

The fibrinolytic capacity of MCs is essential to maintain the production/degradation balance of matrix components to avoid the formation of peritoneal adherences. Treatment of omentum MCs with TGF-β1 inhibited the expression of the fibrinolytic factors tissue-type plasminogen activator (tPA), urokinase-type plasminogen activator (uPA) and uPA receptor (uPAR). Interestingly, treatments with different doses of Tamoxifen restored the basal levels of these factors or even increased their synthesis above basal levels (Figures 7A to 7C). TGF-β1-mediated induction of MMT was also accompanied by increased expression of plasminogen activator inhibitor-1 (PAI-1), a strong anti-fibrinolytic molecule, which was not affected by Tamoxifen (Figure 7D). However, the PAI-1/tPA-ratio, a commonly used marker to determine the decline of fibrinolytic capacity, increased during TGF-β1-induced MMT and returned to basal ratio upon Tamoxifen treatments (Figure 7E).

In contrast, treatment of transdifferentiated MCs from PD effluent with Tamoxifen did not change the PAI-1/tPA-ratio, indicating that these cells retained low fibrinolytic capacity (Figure S3).

### Tamoxifen ameliorates peritoneal alterations induced by dialysis fluid exposure in a mouse PD model

**Figure 8:**
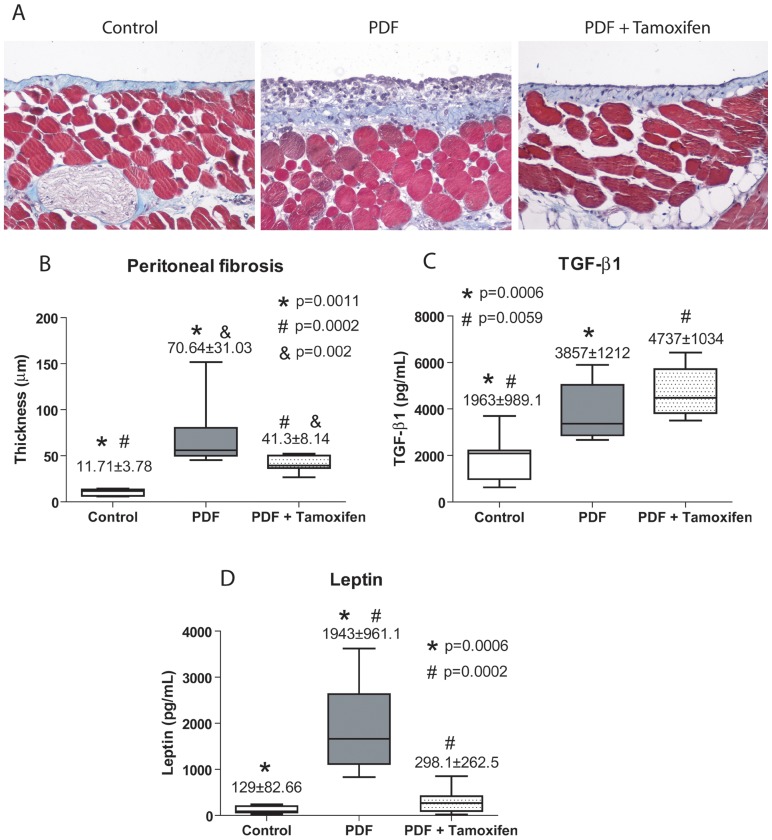
Administration of Tamoxifen decreases PD-induced peritoneal membrane thickness in a mouse model. Mice received a daily instillation of standard PD fluid for 4 weeks with or without the oral administration of Tamoxifen (1.5 mg/kg/day: PDF, n = 14 and PDF + Tamoxifen, n = 17). A control group of mice that were instilled with saline was also included (Control; n = 9). Peritoneal samples were prepared and analyzed as described in the Concise Methods. **(A)** Standard PD fluid exposure increases matrix deposition and the thickness of the peritoneal membrane, while Tamoxifen administration significantly reduces these effects when measured in Masson's trichrome stained sections (representative slides). Magnification ×200. **(B)** The peritoneal thickness ( µm) is increased in DPF group compared with control mice, and the group PDF with Tamoxifen shows a significant reduction of thickness when compared with PDF group. Analysis of variance results in a significance of p<0.0001 (ANOVA). Box Plots graphic represent 25th and 75th percentiles, median, minimum and maximum values of thickness ( µm). **(C)** Measurement of TGF-β1 in the drained volumes shows an increase of this growth factor in PD fluid-instilled animals and administration of Tamoxifen does not reduce TGF-β1 production. The statistical analysis of variance gives a significance of p = 0.0003. **(D)** Measurement of leptin in the drained volumes demonstrates an increase of this adipocytokine in PD fluid-instilled animals and administration of Tamoxifen significantly reduces leptin production, p<0.0001 (ANOVA). Box plots are depicted as picograms per millilitre (pg/mL) and represent the median, minimum and maximum values, as well as the 25th and 75th percentiles. Numbers above boxes depict means ± SE. Symbols represent the statistical differences between groups.

We analyzed whether Tamoxifen might prevent the deterioration of the PM in a mouse model of PD fluid exposure. Histological analysis of parietal peritoneum biopsies from animals exposed to PD fluid (PDF group, n = 14) showed a loss of MCs monolayer and increased PM thickness when compared with mice exposed to saline solution (Control group, n = 9) (Figures 8A and 8B). Oral administration of Tamoxifen (1.5 mg/kg/day) to PD fluid-treated mice (PDF + Tamoxifen group, n = 17) significantly reduced the peritoneal thickness and preserved the mesothelium (Figures 8A and 8B).

It has been described that Leptin, a pro-inflammatory adipocytokine, is able to increase TGF-β1 synthesis and to cooperate with TGF-β1 in the myofibroblast conversion of MCs. We found that Tamoxifen had no effect on TGF-β1 concentrations in the PD effluents but it decreased the Leptin levels (Figures 8C and 8D). These data suggested that Tamoxifen ameliorated peritoneal thickness by impairing TGF-β1-Leptin synergism.

**Figure 9:**
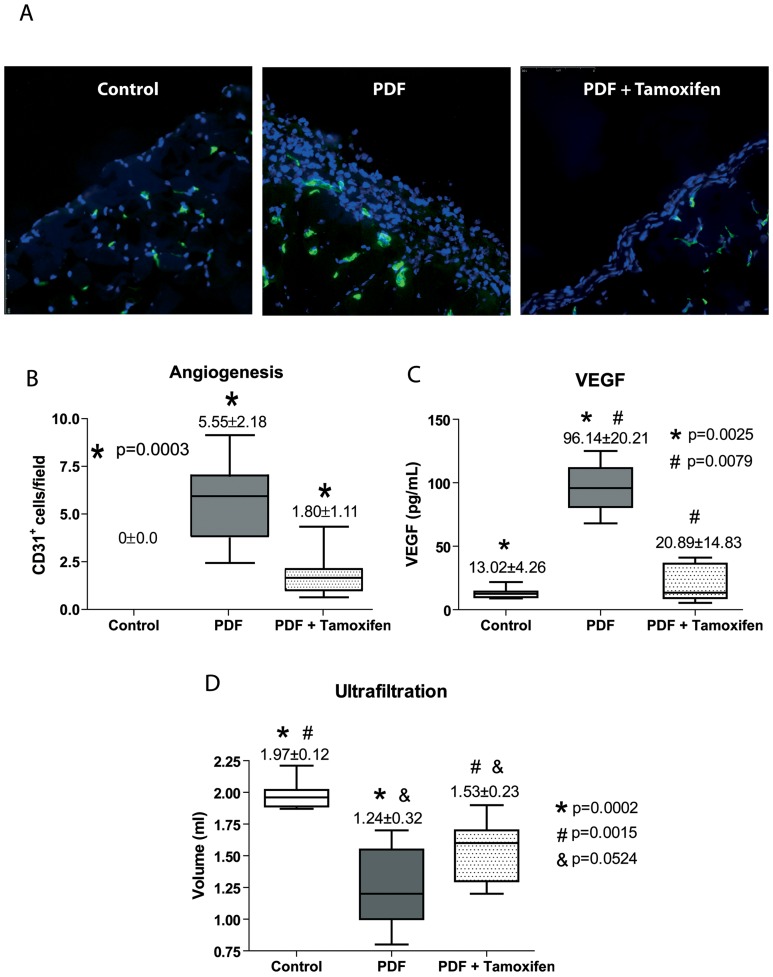
Treatment with Tamoxifen decreases PD-induced angiogenesis, inhibits VEGF production and improves peritoneal ultrafiltration. Mice received a daily instillation of standard PD fluid with or without the oral administration of Tamoxifen (PDF; n = 14 and PDF + Tamoxifen; n = 17). A control group of mice that were instilled with saline was also included (Control; n = 9). **(A)** Standard PD fluid exposure increases peritoneal angiogenesis and Tamoxifen administration significantly reduces the number of vessels, as determined by CD31 staining (representative slides). Magnification ×200. **(B)** Box plots represent the CD31^+^ staining in the different experimental groups and show a decrease of angiogenesis in the Tamoxifen-treated animals. **(C)** Analysis of VEGF in the drained volumes shows a strong increase of this growth factor in PD fluid-instilled animals, and administration of Tamoxifen significantly reduces VEGF production. The ANOVA test resulted in a significance of p<0.0001. **(D)** A 30 minutes ultrafiltration test was performed on the last day of treatments. The volumes recovered from animals exposed to PD fluid are lower than those from mice instilled with saline solution and an increase of net ultrafiltration is obtained in mice exposed to PD fluid that were administrated Tamoxifen. A significance of p<0.0001 was obtained with the analysis of variance test. Box Plots graphics represent 25th and 75th percentiles, median, minimum and maximum values. Numbers above boxes depict means ± SE. Symbols represent the statistic differences between groups.

Angiogenesis is an important process that occurs in the PM during PD, and robust anti-angiogenic effect of Tamoxifen has been described in other tissues. To test the effect of Tamoxifen on PD fluid-induced angiogenesis, blood vessels of the parietal peritoneum were stained with an anti-CD31 antibody. As expected, in the peritoneum from control saline-treated mice the expression of CD31 was confined to deeper vessels located in the muscular tissue (Figure 9A). However, there was a significant increase in the number of submesothelial vessels in PD fluid-instilled mice when compared with the control mice; and administration of Tamoxifen to PD fluid-instilled mice significantly reduced this angiogenesis (Figures 9A and 9B). To further explore the effects of Tamoxifen on angiogenesis, the effluent levels of VEGF were measured in the different experimental conditions. PD fluid exposure strongly increased the concentration of VEGF in the peritoneal cavity, and administration of Tamoxifen significantly reduced the levels of this factor (Figure 9C). The protective effects of Tamoxifen described here had significant implications for the preservation of peritoneal function. Indeed, Tamoxifen treatment partially preserved the ultrafiltration capacity of PD fluid-instilled mice (Figure 9D).

### Tamoxifen reduces PD fluid-induced MMT *in vivo*

**Figure 10:**
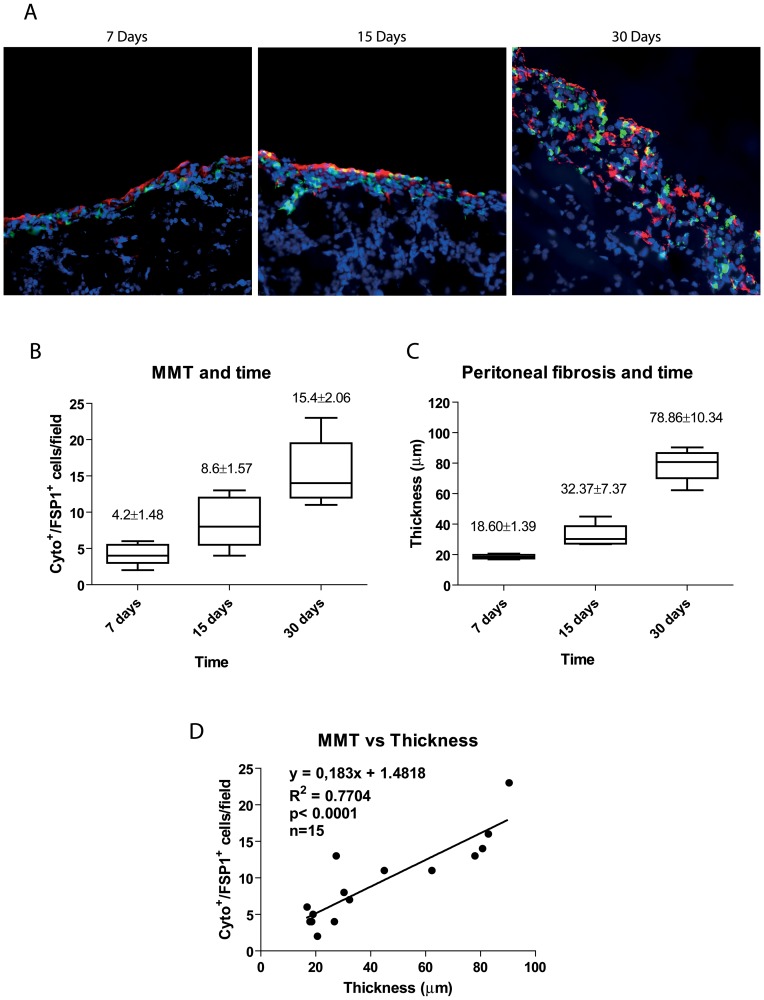
Parallelism between MMT, PM thickness and time on PD. **(A)** Immunofluorescence microscopy images of parietal peritoneal sections stained for cytokeratin (green) and FSP-1 (red), with DAPI counterstaining, show accumulation of trans-differentiated mesothelial cells in the submesothelial space at 7, 15 and 30 days of PD mice. Progressive time-dependent increases of MMT and PM thickness is observed during PD fluid exposure. Representative slides are presented. Magnification ×200. **(B)** Quantification of the submesothelial MMT (cytokeratin/FSP-1 double positive cells per field) at different time points. **(C)** Quantification of peritoneal thickness ( µm) at different time points. Box Plots represent 25% and 75% percentiles, median, minimum and maximum values. Numbers above boxes depict means ± SE. Symbols show statistical differences between groups. **(D)** Correlation between both MMT and peritoneal thickness was determined by Spearman regression analysis.

Another characteristic histological change of the peritoneum during PD is the accumulation of fibroblasts expressing “fibroblast specific protein-1” (FSP-1) in the submesothelial compact zone, some of which co-express cytokeratin, indicating their mesothelial origin via MMT. In order to evaluate the association between MMT and peritoneal fibrosis progression, we analyzed the peritoneal tissue of mice exposed to PD fluid during 7, 15 or 30 days. Both Cyto^+^/FSP-1^+^ staining and peritoneal fibrosis increased in a time-dependent manner (Figures 10A to 10C). Furthermore, MMT and PM fibrosis showed a strong correlation (Figure 10D). These findings indicated that the MMT was not only an early phenomenon that triggered the fibrotic process, but rather it accompanied fibrosis progression until late stages.

**Figure 11:**
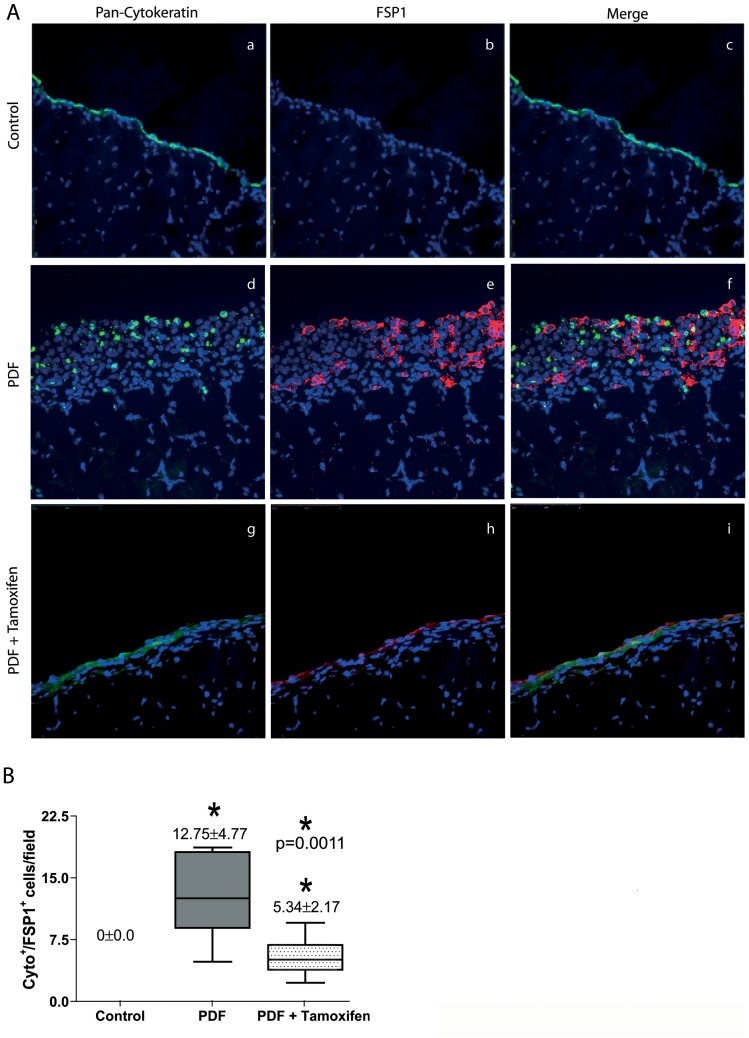
Effects of Tamoxifen on the number of fibroblasts derived from MCs. **(A)** Immunofluorescence microscopy analysis of parietal peritoneal sections stained for cytokeratin (green) and FSP-1 (red) with DAPI counterstaining show accumulation of trans-differentiated mesothelial cells in the submesothelial space (cytokeratin positive cells) in the PDF group, some of which co-express FSP-1 (yellow cells in the Merge panel). The administration of Tamoxifen reduces the number of cytokeratin/FSP-1 double positive cells per field. Representative slides are presented. Magnification ×200. **(B)** Reductions of the number of cytokeratin/FSP-1 positive fibroblasts by Tamoxifen are significant. Box Plots represent 25% and 75% percentiles, median, minimum and maximum values. Numbers above boxes depict means ± SE. Symbols show statistical differences between groups.

Having shown that MMT is a phenomenon that progresses in direct proportion to the time on PD and that Tamoxifen blocked MMT and migration of MCs *in vitro*, we analyzed whether this drug also exerted a MMT-blocking effect *in vivo* in the mouse PD model (four weeks). As expected, in the peritoneum from control saline-treated mice, there was no expression of FSP-1 and the expression of cytokeratin was exclusively restricted to the preserved mesothelium (Figure 11A). By contrast, in the peritoneal tissue of PD fluid-instilled mice there was submesothelial accumulation of FSP-1^+^ fibroblasts, and a percentage of these fibroblasts co-expressed cytokeratin (Figures 11A and 11B). Administration of Tamoxifen to PD fluid-instilled mice significantly reduced the total number of FSP-1^+^ cells, and in particular of the Cyto^+^/FSP-1^+^ subpopulation (Figures 11A and 11B).

## Discussion

Sclerotic peritonitis syndromes (SPS) includes a wide range of peritoneal fibrosis that develops progressively during PD treatment and reaches severe fibrosis in 40 to 70% of cases. SPS has been traditionally considered a reversible condition, while EPS still progresses even after the interruption of PD treatment [4]. Although the pathways to reach EPS from SPS have not been fully established, emerging evidences have indicated that MMT is persistently present in initial and end-stages of peritoneal fibrosis [1], [8], [34]. In this study, we demonstrate that MMT progresses in parallel with peritoneal fibrosis in the mouse PD model. Tamoxifen has been extensively used for the treatment of advanced fibrotic disorders, but there are only few data about his beneficial effect on the prevention or blockade of fibrosis at early stages. In this context, it has been recently shown that Tamoxifen treatment protects from renal fibrosis in a rat model of hypertensive nephrosclerosis [19]. Herein, we explored the effects of Tamoxifen on early stages of peritoneal fibrosis induced by PD.

Given the central role of the MMT process in the initiation and progression of peritoneal injury in PD patients [7], [8], [34], we have analyzed the effects of Tamoxifen on the MMT of MCs *in vitro* and in a mice PD model. We found that Tamoxifen is able to block and to revert the MMT of MCs induced by TGF-β1 and that this drug partially reverts the mesenchymal phenotype of effluent-derived MCs. Although Tamoxifen may also act on cells lacking estrogen receptors [35], herein we confirmed the presence of α-ER in MCs, the expression of which increased in response to administration of Tamoxifen and/or TGF-β1. Tamoxifen was initially described as an anti-estrogen drug, however more recently, in agreement with our results it has been shown that Tamoxifen may also act as an agonist of the α-ER in some cell types, such as endothelial cells [36]. In fact, in transdifferentiated MCs from PD effluent the α-ER is highly expressed and Tamoxifen further increases this expression (Figure 1).

Of note, Tamoxifen blocks TGF-β1-induced down-regulation of E-cadherin and up-regulation of the transcriptional repressors Snail in a context of high expression levels of α-ER (Figures 1 and 4). This is in good agreement with results obtained in breast cancer cells, in which the over-expression of exogenous α-ER induced E-cadherin and down-regulated the expression of Snail, and clones exhibiting these changes grew in clumps and became less invasive [37]. Conversely, when α-ER was knocked down in α-ER-positive breast cancer cell lines, Snail increased, E-cadherin decreased and cells became spindle-shaped and exhibited increased migratory/invasive capacity [37]. In this context, we observed that MCs treated with TGF-β1 acquire increased migratory capacity, and that Tamoxifen treatments reduce this capacity, probably by blocking MMP-2 expression.

As a consequence of its ability to block and to revert the MMT process, Tamoxifen-treatment decreases the production of extracellular matrix components (collagen I and fibronectin) and preserves the fibrinolytic capacity of MCs, which could explain the anti-fibrotic effect exerted by this drug on the PM. Herein, we demonstrate that Tamoxifen ameliorates peritoneal thickness and decreases submesothelial accumulation of transdifferentiated MCs in PD fluid-instilled mice. Our results suggest a dual anti-fibrotic effect of Tamoxifen on MMT *in vivo*: 1.- direct blocking of the MMT process itself, and 2.- inhibition of leptin expression, and therefore impeding the TGF-β1-leptin cooperation in the induction of MMT and fibrosis. In this context, it has been shown that peritoneal adipocytes exposed to glucose from PD liquids produce leptin, which in turn promotes TGF-β1 production by MCs and cooperates with TGF-β1 in the induction of MMT of MCs [38], [39]. In concordance with our results, it has recently been reported that Tamoxifen can reduce circulating levels of leptin [40], although this results remains to be confirmed [41], [42], seems clear that leptin is a key molecule in the induction MMT, tissue fibrosis and angiogenesis. Another possible anti-fibrotic mechanism associated with Tamoxifen could be the down-regulation of the pro-fibrotic connective tissue growth factor (CTGF) [17].

Angiogenesis is an important sign of type-I PM failure and we demonstrate that Tamoxifen decreases the number of submesothelial vessels in mice peritoneal tissue exposed to PD fluid, which may be due to Tamoxifen-mediated down-regulation of VEGF expression in the effluents. This anti-angiogenic effect could also be due to a reduction of leptin levels, given that both molecules may act in a cooperative manner to induce neovascularization [43]. Importantly, this vessels reduction resulted in the maintenance of the UF capacity reinforcing the idea that increase in peritoneal vessels number are key in the hyperfiltration associated to type-I PM failure.

The robustness of our results in both *in-vivo* and *in-vitro* together with previous clinical studies invite to use Tamoxifen in SPS to prevent the type-I PM failure. Our clinical experience with Tamoxifen for the treatment of EPS started in 1992 after the positive results of Clark et al. using Tamoxifen to treat retroperitoneal fibrosis [14]. We demonstrated a significant improvement in survival and a reduction of abdominal complications in nine patients with EPS orally treated with Tamoxifen [15]. Afterwards we extended our study by including five additional patients and by expanding the time of follow-up, which confirmed the beneficial effects of Tamoxifen in terms of abdominal complications, hospital admissions and mortality (data not shown). Recently, Korte et al. published the results of the Multicenter Dutch EPS study, which concluded that almost from the start of the study the group treated with Tamoxifen showed better survival than non-treated group [16]. Thus, there is increasing evidence about the benefic effect of Tamoxifen for treating retroperitoneal fibrosis and EPS alone or in combination with immunosuppressant drugs [12].

In conclusion, it is tempting to hypothesize that MMT, peritoneal fibrosis and EPS may be part of the same patho-physiologic process and therefore, Tamoxifen exerts a protective effects on the peritoneal membrane during both early and late stages of fibrotic disorders. Following exposure to PD fluids, Tamoxifen protects the peritoneum by inhibiting the production of matrix components, leptin, and VEGF, and maintaining the fibrinolytic capacity. Together these effects, Tamoxifen contribute to the anti-MMT, anti-fibrotic and anti-angiogenic activities observed.

## Supporting Information

Figure S1 **Effect of Tamoxifen on MCs apoptosis** ***in vitro*** **.** MCs were cultured to subconfluence and rested for 24 hours. The cells were pre-incubated with Tamoxifen (3, 6 and 10 µM) for one hour and treated with 100 nM Staurosporine (STS) for 24 hours. Flow cytometry assessment of DNA content shows that treatment with different doses of Tamoxifen decreases apoptosis of cells treated with STS. Bars depict the means ± SD of three independent experiments performed in triplicate and the symbol represents the statistical differences.
(TIF)

Figure S2 **Effect of Tamoxifen on the wound repair capacity of MCs.** Omentum-derived MCs, administered Tamoxifen (3 and 6 µM) or not, were subjected to mechanical injury and photographed every twelve hours over 72 hours. MCs treated with 6 µM of Tamoxifen showed a slight delay at 24 and 48 hours in closing the wound. However, at 72 hour there were minimal differences between cells treated or not with Tamoxifen.
(TIF)

Figure S3 **Tamoxifen does not modify the PAI/tPA-ratio in transdifferentiated MCs from PD effluent.** Non-epitheliod effluent-derived MCs were treated with Tamoxifen (3, 6 and 10 µM) during 48 h. The levels of PAI-1 and tPA were measured in culture media supernatants by ELISA. Results show that Tamoxifen does not modify the PAI-1/tPA-ratio.
(TIF)

Table S1 **Effect of Tamoxifen on cellular Cycle of HPMC (% gated).**
(DOC)
