# Supplementary figures and images for: Tamoxifen Ameliorates Peritoneal Membrane Damage by Blocking Mesothelial to Mesenchymal Transition in Peritoneal Dialysis
Source: PLoS One. 2013 Apr 23;8(4):e61165. doi: 10.1371/journal.pone.0061165 (PMC3634067; doi:10.1371/journal.pone.0061165)

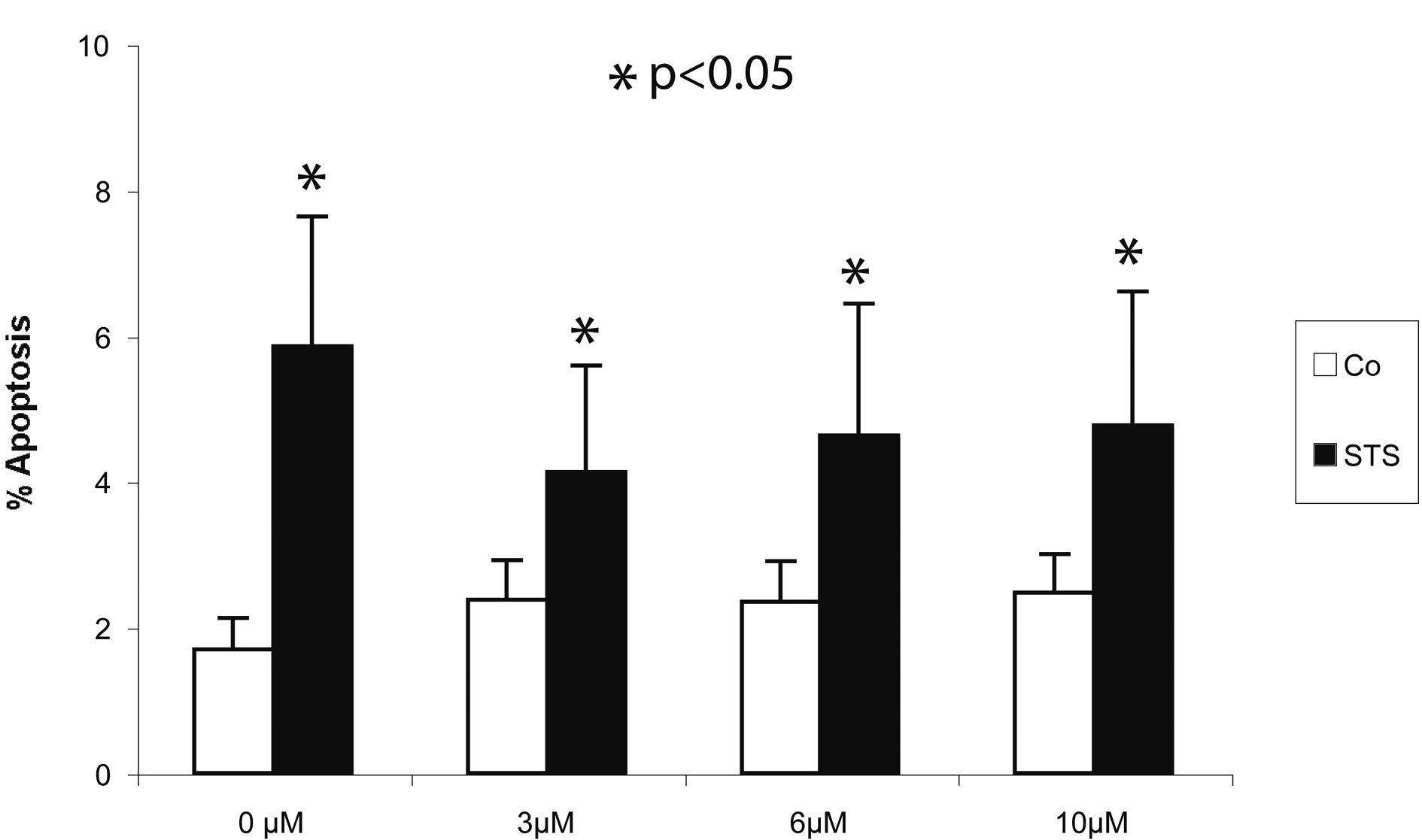

Supplement: Figure S1 — Effect of Tamoxifen on MCs apoptosis in vitro . MCs were cultured to subconfluence and rested for 24 hours. The cells were pre-incubated with Tamoxifen (3, 6 and 10 µM) for one hour and treated with 100 nM Staurosporine (STS) for 24 hours. Flow cytometry assessment of DNA content shows that treatment with different doses of Tamoxifen decreases apoptosis of cells treated with STS. Bars depict the means ± SD of three independent experiments performed in triplicate and the symbol represents the statistical differences. (TIF) [file pone.0061165.s001.tif]

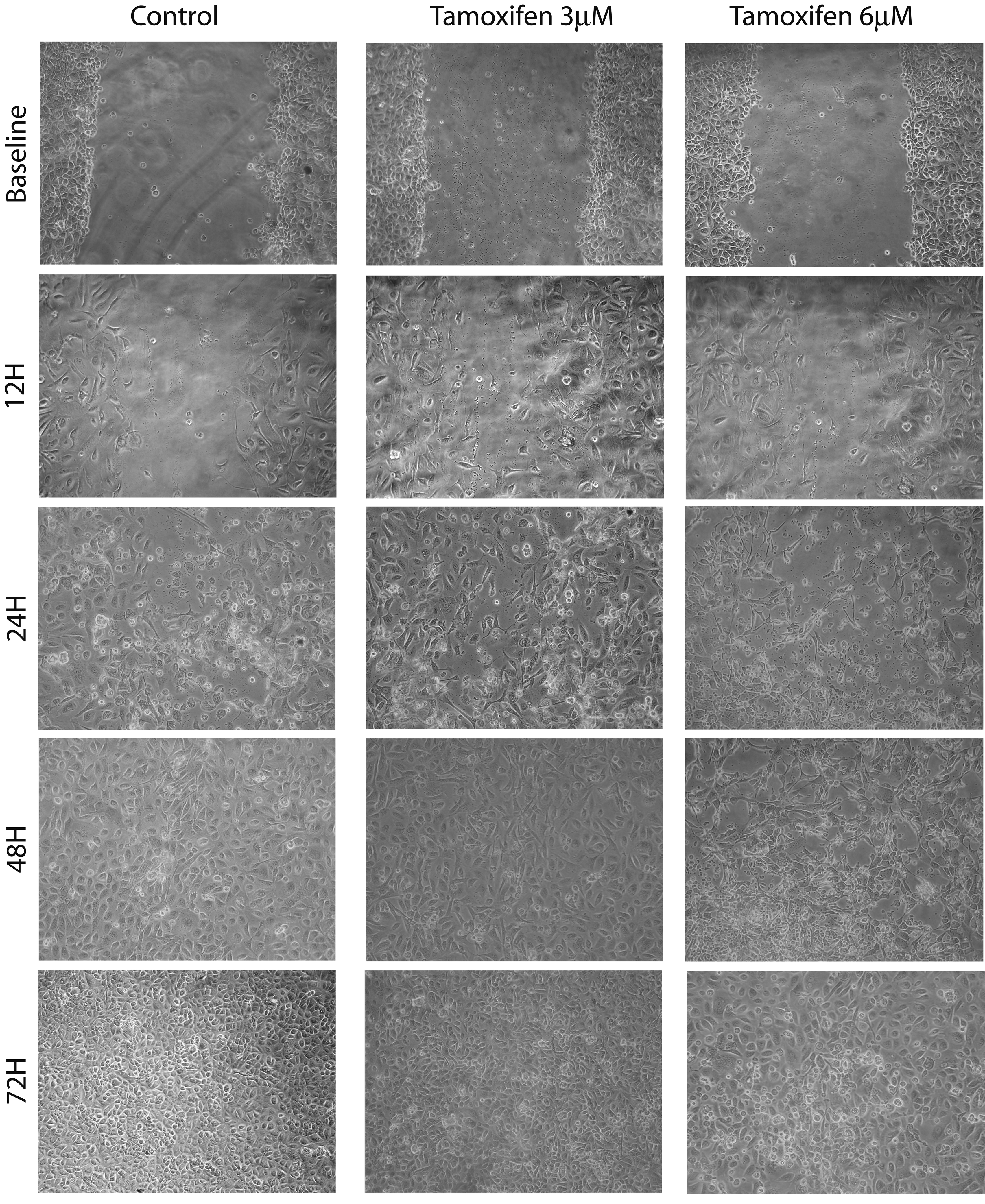

Supplement: Figure S2 — Effect of Tamoxifen on the wound repair capacity of MCs. Omentum-derived MCs, administered Tamoxifen (3 and 6 µM) or not, were subjected to mechanical injury and photographed every twelve hours over 72 hours. MCs treated with 6 µM of Tamoxifen showed a slight delay at 24 and 48 hours in closing the wound. However, at 72 hour there were minimal differences between cells treated or not with Tamoxifen. (TIF) [file pone.0061165.s002.tif]

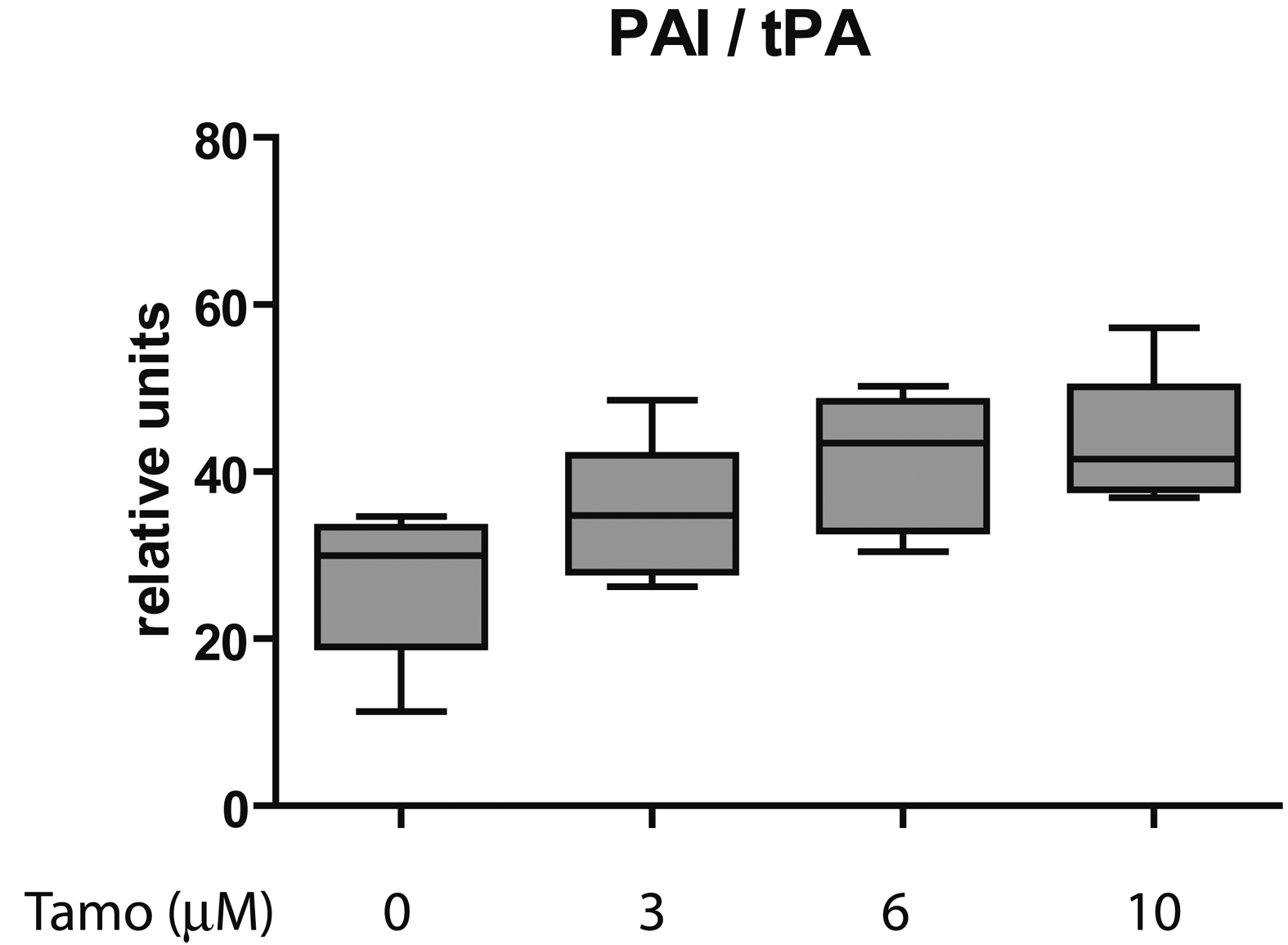

Supplement: Figure S3 — Tamoxifen does not modify the PAI/tPA-ratio in transdifferentiated MCs from PD effluent. Non-epitheliod effluent-derived MCs were treated with Tamoxifen (3, 6 and 10 µM) during 48 h. The levels of PAI-1 and tPA were measured in culture media supernatants by ELISA. Results show that Tamoxifen does not modify the PAI-1/tPA-ratio. (TIF) [file pone.0061165.s003.tif]
