# Supplementary material for: Tamoxifen Ameliorates Peritoneal Membrane Damage by Blocking Mesothelial to Mesenchymal Transition in Peritoneal Dialysis
Source: PLoS One. 2013 Apr 23;8(4):e61165. doi: 10.1371/journal.pone.0061165 (PMC3634067; doi:10.1371/journal.pone.0061165)
Supplement: Table S1 — Effect of Tamoxifen on cellular Cycle of HPMC (% gated). (DOC) [file pone.0061165.s004.doc]

Supplementary table S1. Effect of Tamoxifen on cellular Cycle of HPMC (% gated).

| **Cell Cycle** | **Control** | **Tamo 3 μM** | **Tamo 6 μM** | **Tamo 10 μM** |
| --- | --- | --- | --- | --- |
| **Resting cells (M1)** | 75.9 | 76.07 | 76.14 | 77.32 |
| **DNA Division (M2)** | 5.7 | 5.1 | 5.3 | 4.91 |
| **New cell synthesis (M3)** | 14.4 | 14.8 | 14.9 | 14.14 |
| **Apoptosis (M4)** | 4.0 | 4.03 | 3.66 | 3.63 |
